# Torrefaction of kraft pulp mills sludges

**DOI:** 10.1038/s41598-023-46158-0

**Published:** 2023-12-14

**Authors:** Caio Moreira Miquelino Eleto Torres, Angélica de Cássia Oliveira Carneiro, Bruna Virgínia Cunha Rodrigues, Marina Foresti Salgado Bravo, Claudio Mudadu Silva

**Affiliations:** 1 Departamento de Engenharia Florestal, Universidade Federal de Viçosa, Av. Peter Henry Rolfs, Campus UFV, Viçosa, Minas Gerais 36.570-900 Brazil; 2 Departamento de Engenharia Civil, Universidade Federal de Viçosa, Av. Peter Henry Rolfs, Campus UFV, Viçosa, Minas Gerais 36.570-900 Brazil

**Keywords:** Environmental sciences, Chemistry, Engineering

## Abstract

Torrefaction emerges as an industrial process that increases the energy content of conventional biomass. Primary and secondary sludge are the main solid residues generated in the Effluent Treatment Plants of bleached kraft pulp mills, and can be considered as biomass. Typically, these wastes are sent to industrial landfills. The present study aimed to evaluate the technical feasibility of transforming the primary sludge (PS), secondary sludge (SS) and mixed sludges (MIX) into torrefied biomass for energy generation. Three temperatures (260, 290 and 320 °C) and three residence times (20, 40 and 60′) were used in the sludge torrefaction process. Increasing the torrefaction temperature and residence time of the sludges produced several benefits on their physical and chemical properties. They promoted an increase in the heating value, due to the elimination of less energetic compounds and the concentration of the fixed carbon content; caused a reduction of moisture, with a consequent increase in the lower heating value of the sludges; and led to a high energy yield and an increased energy density, important parameters in sludges energy generation. The treatment at 320 °C for 60′ obtained increases of 76%, 27% and 41% over the reference, for PS, SS and MIX, respectively.

## Introduction

Renewable energy has emerged in recent years as an important driver of economic and social development to help protect communities from the effects of climate change^1^. Bioenergy is a valuable component of the renewable energy mix and can play a role in meeting our energy needs^2,3^.

Biomass can be sourced from planted forests or derived from wood residues, encompassing both forestry and industrial sectors, thus serving as a valuable renewable resource for energy production^4–6^. The transformation of these co-products into a biomass with a higher energy density and heating value can be obtained through the torrefaction process^7^.

Primary and secondary sludges are the main solid wastes generated in the Effluent Treatment Plants (ETP) of bleached kraft pulp mills, and can be regarded as significant biomass resources within the forest industry. According to Brazilian Technical Standard ABNT NBR 10004/2004, sludge is classified as Class II A, non-hazardous and non-inert^8–10^.

Primary sludge comes from the primary treatment of effluents and has a high fiber content. Secondary sludge comes from biological treatment (activated sludge) and consists mainly of microorganisms.

In several pulp mills, secondary sludge is mixed with primary sludge to facilitate water removal in the sludge dewatering process, which is normally carried out by belt presses or other pressing mechanism. An increase in dry solids from 2–3 to 25–40% can be accomplished^11^. Kraft mills produce, on average, 58 kg per air-dried ton of pulp (kg adt^−1^), of which 70% refers to primary sludge and 30% to secondary sludge^12,13^. In 2022, Brazilian pulp mills generated approximately 1 million tons of primary sludge and 375 thousand tons of secondary sludge^14^.

In Brazilian pulp mills, industrial landfilling is still the predominant practice for the disposal of both sludges. However, this approach can cause negative environmental impacts, such as soil and groundwater contamination. In addition, this approach has other negative aspects, such as high costs and increasingly stringent legislation. Costs can range from $30 per ton of sludge. The financial burden of sludge management is significant for these plants, with approximately 60% of total effluent treatment costs allocated to sludge management and disposal^15–17^.

The energetic content of sludge is comparable to wood and other biomasses that are already frequently used for energy generation. In the search for an increase in the energy potential of sludge, torrefaction emerges as an alternative capable of producing biomass with a lower hygroscopicity, a higher heating value and high carbon content^18–20^. In their respective research, Doddapaneni (2022) and Huang (2017) found significant increases in the higher heating value of sludge after torrefaction at 300 °C, with increases of 19% and 50%, respectively^21,22^.

Torrefaction is a versatile technique that can be employed on a wide variety of biomass^18,19,21^. To ensure better suitability and performance of torrefied materials, the process variables need to be properly defined and optimized. A gap was observed in parameters such as the optimal torrefaction temperatures and residence times. These parameters directly affect the mass balance, the quality and the final cost of the roasted products obtained from the ETP sludge from kraft pulp mills.

This approach holds promise for addressing the environmental, social, and governance (ESG) criteria, sustainable development goals (SDGs), and circular economy challenges facing the industry.

In the current literature, no technical scientific study was found showing the feasibility for the use of primary and secondary sludge generated in ETP of bleached kraft pulp mills as the raw material for torrefaction. Biomass torrefaction is not a new thermal treatment method. However, the use of this method for kraft pulp mill sludges has never been reported. The production of torrefied sludges proved to be an attractive alternative. Most kraft bleached pulp mills have biomass boilers, suitable for the application of this modern and sustainable biomass that contributes to energy efficiency with environmental and economic gains.

This study aimed to evaluate the technical feasibility of transforming primary sludge, secondary sludge and mixed sludge (the mixture of primary and secondary sludges) generated in a ETP of a Brazilian bleached kraft pulp mill into torrefied biomass for energy generation by optimizing the process parameters.

## Material and methods

The primary sludge (PS) and secondary sludge (SS) were obtained from the ETP of a Brazilian bleached kraft pulp company. The experiment was developed in the Pulp and Paper Laboratory (LCP) and the Wood Panels and Energy Laboratory (LAPEM), both linked to the Forest Engineering Department of the Federal University of Viçosa, in Viçosa, Minas Gerais, Brazil.

The percentage of the mixture of sludges in the mixed sludge (MIX) (75% primary sludge and 25% secondary sludge) was based on the proportion usually generated at the mill.

The sludge samples were stored separately in polyethylene bags with a moisture content, on a wet basis, of 61 ± 1% for the PS, 86 ± 1% for the SS and 72 ± 1% for the MIX. They were placed in a cold storage room at a temperature of 6 ± 2 °C to avoid contamination and maintain their preservation.

**Figure 1:**
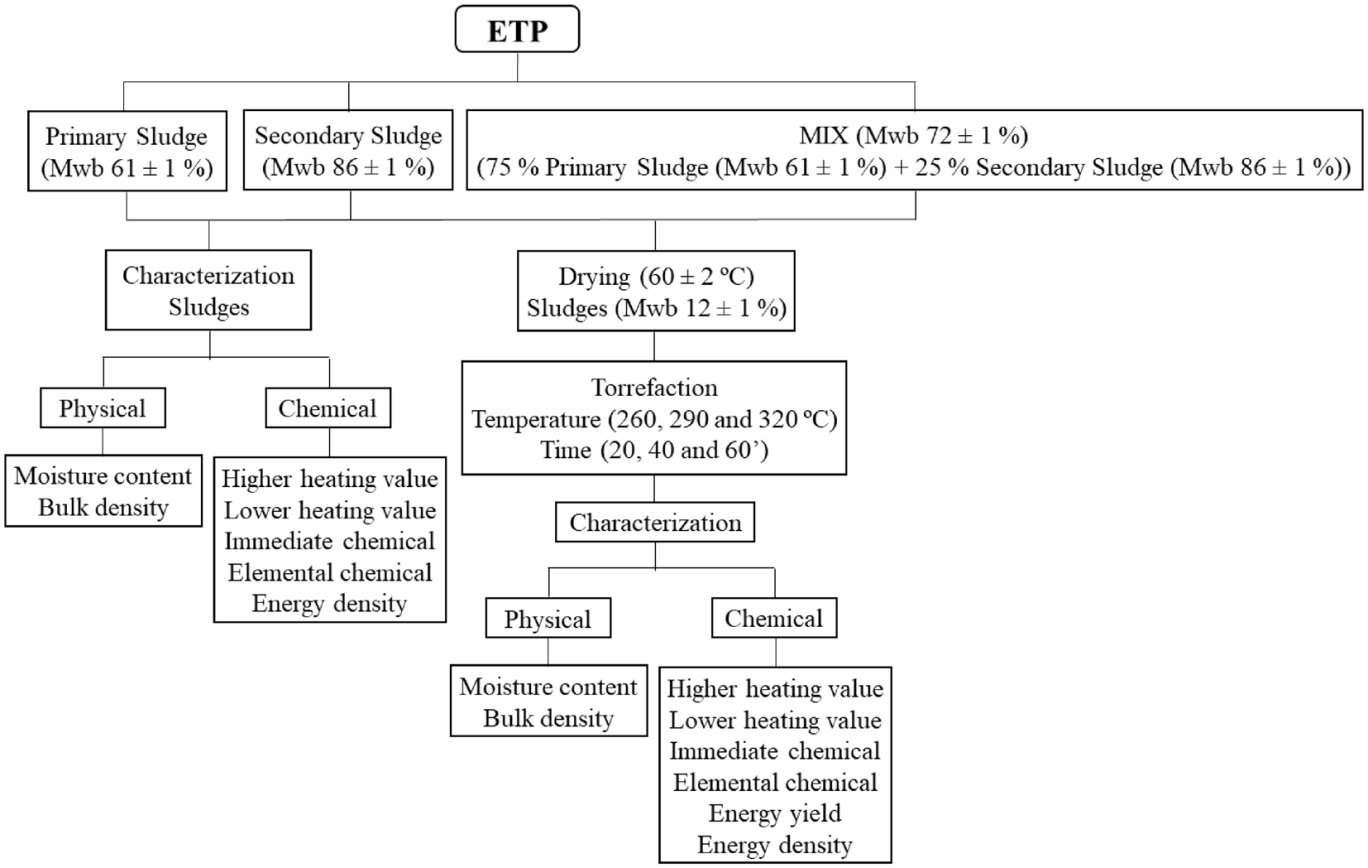
Schematic flowchart of the experimental plan.

The sludges were subjected to the experimental script described by the flow chart in Fig. 1.

The term "reference sludge" refers to sludge samples that have not undergone any thermal or chemical treatment, serving as a reference for comparison with samples that have undergone thermal treatments. These reference samples are important to evaluate the effect of heat treatments on the chemical composition of the sludge and to determine the energy potential of treated sludge compared to sludge without heat treatment.

The PS, SS and MIX were characterized reference, that is, at the outlet of the ETP, by determining the moisture content, higher heating value and bulk density.

The sludges were dried in a SOLAB model SL102/1000 oven, with air circulation and renewal, at a temperature of 60 ± 2 °C, until reaching a moisture content, on a wet basis, of 12 ± 1%. After drying, each sludge was characterized to obtain its physical (moisture content and bulk density) and chemical (heating value—higher and lower, chemical—immediate and elemental and energy density) properties.

The secondary sludge, after drying, formed rigid and irregular agglomerates. To promote their homogenization, it was necessary to grind them. This characteristic is due to the fact that it is a fine material, generally non-mineralized, formed mainly by microorganisms and complex biopolymers, such as lignocellulosic compounds^13,23,24^.

The torrefied products of the PS, SS and MIX were characterized as to obtain its physical (moisture content and bulk density) and chemical (heating value—higher and lower, chemical—immediate and elemental, energy yield and energy density) properties.

### Sludge properties

The moisture content of the sludges was obtained according to European Standard EN 14774-1^25^. The bulk density (kg/m^3^) of the sludges was obtained according to Standard EN 15103^26^.

The ash content of the sludges was obtained according to Standard EN 14775^27^. The volatile matter of the sludges was obtained according to Standard EN 15148^28^. The fixed carbon was calculated using the sum of the ash and volatile matter content decreased by 100.

The percentages of the elemental chemical composition (carbon, hydrogen, nitrogen, and sulfur) of the sludges were determined using Elemental Analyzer equipment, model TruSpec CHN Micro and TruSpec S. The oxygen value was obtained by adding carbon, nitrogen, hydrogen, sulfur and ash content, decreased by 100, according to Standard EN 15296^29^.

The higher heating value was determined using a Parr 6300 adiabatic calorimeter, based on the American Society for Testing and Materials Standard D2015^30^.

The estimation of the lower heating value was performed using Eq. 1, according to Annex E of Standard EN 14918^31^.

1 $${\text{LHV}}\left( {_{{\text{constant pressure}}} } \right) = \left( {{\text{HHV}}{-}{212}.{2} \times {\text{H}}{-}0.{8} \times \left( {{\text{O}} + {\text{N}}} \right)} \right) \times \left( {{1}{-}0.0{1} \times {\text{M}}} \right) - \left( {{24}.{43} \times {\text{M}}} \right)$$

where LHV (_constant pressure_): lower heating value at constant pressure, in J g^−1^; HHV: higher heating value, in J g^−1^; H, O, N: hydrogen, oxygen, and nitrogen, respectively, in percent (%); M: moisture, wet basis, in percent (%).

The enthalpy of vaporization (constant pressure) for water at 25 °C is 44.01 kJ mol^−1^. This corresponds to 218.3 J g^−1^ for 1% (m m^−1^) hydrogen in the sample or 24.43 J g^−1^ for 1% (m m^−1^) moisture, respectively.

The energy density (GJ m^−3^) was obtained by the product of the lower heating value and the bulk density.

### Sludge torrefaction

**Figure 2:**
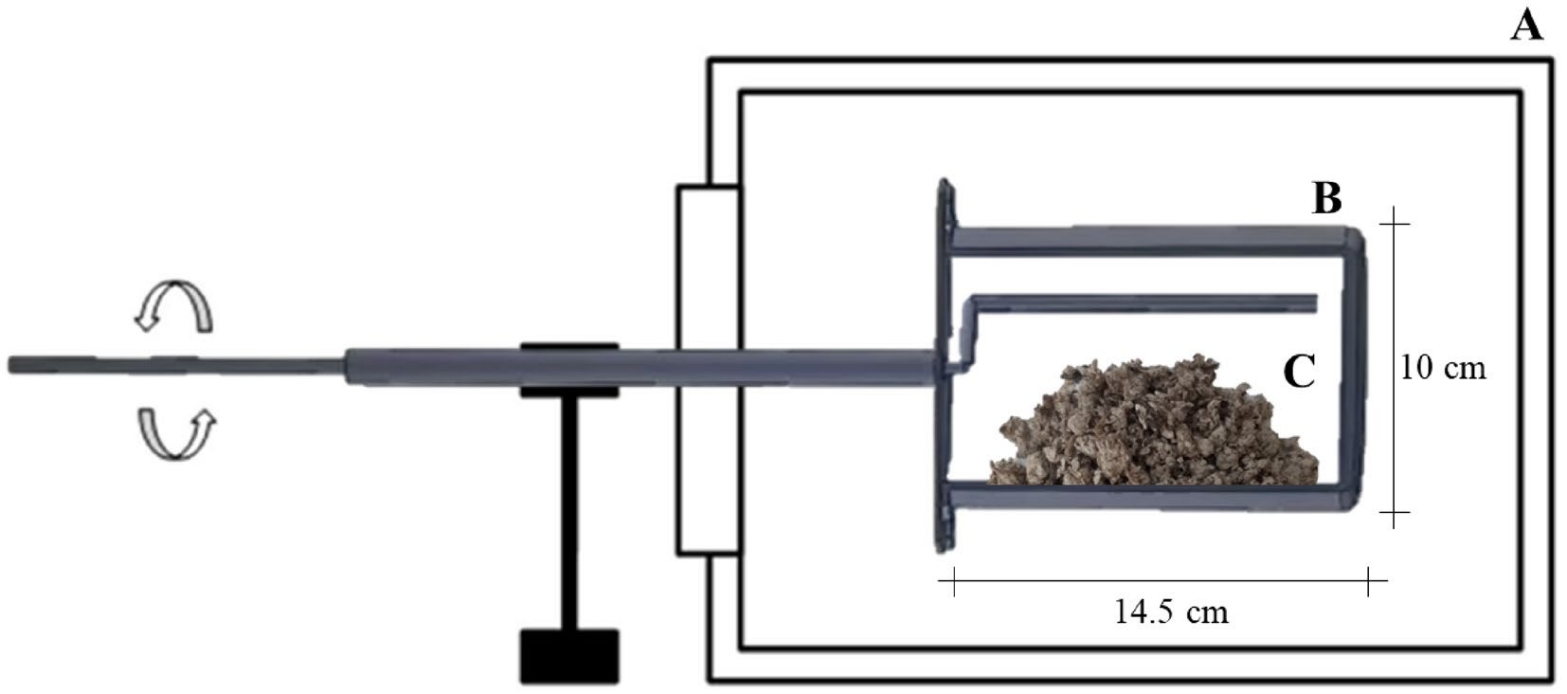
Sludge thermal treatment layout. Muffle (**A**); Container (**B**) and Sludge (**C**).

Samples of 132 ± 2 g of sludge were placed inside a metal container to conduct the thermal treatment for the torrefaction of the sludge. The dimensions of the metal container were 10 cm in diameter and 14.5 cm in height, with a volumetric capacity of 1300 ml, equipped with a lid which permitted the exit of gases resulting from thermal degradation (Fig. 2B).

The container was inserted in an electric oven, an oven muffle with ramps model SSFMr 16 L, connected to the external environment through a hollow metal rod 38 cm long, allowing its constant movement and the release of volatile compounds from the partial thermal degradation of the sludge (Fig. 2).

The container remained in the oven for five minutes until it reached the pre-set torrefaction temperature according to the treatment. After this period, the thermal treatment residence time was counted. The sludge was agitated inside the container every ten minutes, through the use of the hollow metal rod, in order to homogenize the sample.

Three temperatures (260, 290 and 320 °C) and three residence times (20, 40 and 60 min) were used in the sludge torrefaction process. These values were obtained from tests that were performed preliminarily. The temperature was recorded through a datalogger attached to the oven muffle. A portable digital chronometer was used to reference the residence time of the sludges.

After the thermal treatment, the container was removed from the oven muffle and the opening for releasing the gases from the thermal degradation of the sludge was sealed to stop the reactions. The container was placed on a lab bench, at room temperature, until it reached a temperature of approximately 25 °C.

After cooling, the torrefied sludge was removed from the container and weighed to obtain the mass balance of the process, then stored in sealed polyethylene bags for further characterization.

### Efficiency of the torrefaction process

The mass yield of the torrefied sludge was obtained by dividing the final particle mass of the torrefied sludge by the initial particle mass of the reference sludge, multiplied by 100, according to Eq. 2. The mass loss was obtained by the difference between 100 and the mass yield

2 $${\text{MY}} = \left( {{\text{FM}}_{{{\text{ts}}}} /{\text{IM}}_{{\text{s}}} } \right) \times {1}00$$

where MY: mass yield, in %; FM_ts_: final mass of torrefied sludge particles, in kg; IM_s_: initial particle mass of the reference sludge, in kg.

The energy yield was obtained by dividing the average value of the higher heating value (HHV) of the torrefied sludge by the average value of the reference sludge particles HHV multiplied by the mass yield, according to Eq. 3.

3 $${\text{EY}} = \left( {{\text{HHV}}_{{{\text{st}}}} /{\text{HHV}}_{{\text{s}}} } \right) \times {\text{MY}}$$

where EY: energy yield, in %; HHV_st_: higher heating value of the torrefied sludge particles, in kcal/kg; HHV_s_: higher heating value of the reference sludge particles, in kcal/kg; MY: mass yield, in %.

The energy density (GJ m^−3^) of the sludge was obtained by multiplying the lower heating value by the bulk density.

### Experimental design

The experimental design used for statistical analysis was entirely randomized, arranged in a factorial design with two factors: three times (20, 40 and 60 min) and three temperatures (260, 290 and 320 °C), in four repetitions, plus the reference, totaling 37 sampling units for each sludge. The sludges were evaluated independently.

The data (moisture content, bulk density, chemical—elemental and immediate, heating value—higher and lower and energy density) were subjected to analysis of variance (ANOVA), and when there were significant differences between treatments, the Tukey test was applied for the treatments at 5% significance. The results considered to be reference values were submitted to the *Dunnett* test for the treatments at a 5% significance level.

Statistical analyses were performed using SigmaPlot 14.0 (Systat Software, Inc.) and RStudio (R Core Team, 2020) software.

### Complies with international, national and/or institutional guidelines

The experimental research complied with relevant institutional, national, and international guidelines and legislation.

## Results and discussion

### Properties of wet sludge

The lower heating value, moisture contents, bulk and energy density of the PS, SS and MIX, before the drying process, after dewatering presses at the Effluent Treatment Plants (ETP) are presented in Table 1.

**Table 1:** Average values and their standard deviation from the wet sludge properties.

Properties	Sludge		
	Primary	Secondary	MIX
Lower heating value (MJ kg^−1^)	4.84 ± 0.01	0.08 ± 0.07	2.65 ± 0.02
Moisture—wet basis (%)	60 ± 0	86 ± 0	72 ± 1
Bulk density (Kg m^−3^)	419 ± 17	978 ± 10	683 ± 13
Energy density (GJ m^−3^)	2.03 ± 0.01	0.08 ± 0.07	1.81 ± 0.02

The sludge presents a low LHV due to the high moisture content. The SS showed the lowest LHV when compared to the PS and MIX, which is directly related to its higher moisture content. The combustion of sludge in a biomass boiler, when it presents a high moisture content, can result in a zero or even negative energy balance. It is estimated that the energy yield of reference sludge is 35% of its total energy content^13,32,33^.

The bulk density is a factor that generally contributes positively to the increase in energy density. However, it is important to note that the energy density is influenced by the lower heating value and the bulk density of the material, both affected by the moisture content^34,35^. In the case of sludges, the high bulk density is directly related to its high moisture content. This results in a low energy density of the reference sludge, which is a negative point regarding energy reuse^36^.

### Properties of the torrefied sludge

#### Physical analysis

The moisture content, wet basis, and bulk density of both the reference and torrefied samples of PS, SS and MIX are presented in Table 2.

**Table 2:** Average values of moisture content, wet basis, and bulk density of the reference and torrefied sludge samples.

Sludges	Temperature	Moisture—wet basis (%)						Bulk density (%)					
		Time						Time					
		20′		40′		60′		20′		40′		60′	
PS	260 °C	4.3	Ab	1.7	Bc	1.1	Cc	165.9	Ab	149.8	Bb	136.5	Cc
	290 °C	2.6	Ac	1.8	Bc	1.4	Cb	159.1	Ac	147.5	Bbc	140.7	Cb
	320 °C	1.6	Bd	1.9	Ab	1.1	Cc	148.4	Ad	144.9	Bc	103.0	Cd
	Reference	11.6						186.3					
SS	260 °C	3.4	Ab	2.3	Bb	1.0	Cb	603.3	Aa	593.4	Ba*	597.4	Aba*
	290 °C	3.0	Ac	1.1	Bc	0.6	Cc	596.8	Aab*	586.8	Bb	592.5	Aba*
	320 °C	2.2	Ad	1.1	Bc	0.3	Cc	588.3	Ab*	584.8	Ab	586.7	Ab*
	Reference	11.9						592.0					
MIX	260 °C	2.1	Ab	0.9	Bb	0.6	Cb	192.7	Ab	193.5	Ac	182.1	Bc
	290 °C	1.4	Ac	0.9	Bb	0.5	Cb	191.7	Bb	196.0	ABbc	196.4	Bb
	320 °C	1.5	Ac	1.3	Bb	0.7	Cb	194.3	Ab	197.6	Ab	170.8	Bd
	Reference	12.3						218.6					

Averages followed by the same capital letter between lines (time and properties) and lowercase between columns (temperature and sludge) do not differ, at 5% significance, by the Tukey test, for each type of sludge. *Do not differ from the reference, at 5% significance, by the Dunnet test, for each type of sludge.

*PS*—primary sludge, *SS*—secondary sludge, *MIX*—primary and secondary sludge.

The moisture content of the sludge decreased significantly with increasing torrefaction time and temperature, with a reduction of more than 90% compared to the references when thermal treated at 320 °C for 60 min. The lower moisture of the sludge is related to the elimination of some more hydrophilic chemical components, such as hemicelluloses, which are rich in hydroxyl groups (–OH), and the concentration of more thermally stable hydrophobic components^13,37,38^. The moisture loss of torrefied sludge contributes to its low biodegradability and the non-generation of mold, facilitating its storage for long periods of time.

The bulk density of the PS and MIX decreased with increasing temperature and torrefaction time compared to the reference. The reduction in bulk density of the sludge occurred due to moisture loss and as a result of thermal degradation of its main components. In the treatment, at 320 °C for 60 min, with a decrease of 45% and 22% for the PS and MIX, respectively. The bulk density of the reference secondary sludge did not differ for the treatments at 260 °C for 40 min and 60 min; at 290 °C for 20 min and 60 min; and at 320 °C for 20 min and 60 min.

The lignocellulosic composition of sludge with increasing torrefaction time and temperature preserves the most hydrophobic component, mainly the compounds derived from lignin^13,21^. SS and MIX have higher concentrations of hydrophobic components, thus having higher moisture losses during thermal treatments when compared to PS.

PS and MIX had a greater decrease in bulk density because their feedstock comes mainly from lignocellulosic materials with a higher concentration of sugars, such as hemicellulose, which is the fraction most degraded during torrefaction^9,21^.

In general, the bulk density of the thermal treatments of the secondary sludge remained similar to the reference sludge. There was a reduction in the particle size of the sludge after grinding for its homogenization, which promoted an increase in surface area and, consequently, a greater accommodation of the particles. Moreover, this fact shows that there were no modifications or losses by thermal degradation in the sludge composition, probably due to its nature. The secondary sludge is fine and consists essentially of microorganisms and complex biopolymers, such as lignin^23,39^.

#### Analysis of immediate and elemental chemical composition

The average values of the immediate chemical composition (ash content, fixed carbon and volatile matter) of the reference and torrefied sludge samples are presented in Table 3.

**Table 3:** Average values of the immediate chemical composition (ash content, fixed carbon and volatile matter) of the reference and torrefied sludges.

Sludges	Temperature	Ash (%)						Fixed carbon (%)						Volatile matter (%)					
		Time						Time						Time					
		20′		40′		60′		20′		40′		60′		20′		40′		60′	
PS	260 °C	1.7	Bb*	1.8	Bc	2.0	Ac*	11.3	Bab*	11.2	Bbc*	13.0	Ac*	87.0	Aab*	87.0	Aa*	85.1	Aa*
	290 °C	1.7	Cb*	2.2	Bb	2.6	Ab	12.0	Bab*	13.0	Bb	22.1	Ab	86.3	Aab*	84.8	Bb	75.3	Cb
	320 °C	2.0	Ba	2.3	Ba	4.6	Aa	12.7	Ca	19.8	Ba	46.8	Aa	85.3	Ab	77.9	Bc	48.6	Cc
	Reference	1.6						10.7						87.7					
SS	260 °C	25.7	Ba	25.6	Bb	27.0	Ac	11.6	Aab*	10.9	Bab*	12.9	Ab	62.7	Ab	63.5	Ab	60.1	Bb
	290 °C	26.4	Ca	27.7	Ba	29.6	Ab	11.5	Ba	12.3	Ba	14.8	Aa	62.1	Ab	60.0	Bc	55.6	Cc
	320 °C	25.9	Ca	27.6	Ba	32.1	Aa	11.6	Ba	12.3	Ba	16.2	Aa	62.5	Ab	60.1	Bc	51.7	Cd
	Reference	24.2						9.5						66.2					
MIX	260 °C	7.5	Aa*	7.3	Ab*	8.6	Ab*	8.6	Bc	9.9	Bb*	13.2	Ac	83.9	Aa*	82.8	Aa*	78.2	Bb
	290 °C	8.1	Aa*	7.9	Ab*	8.0	Ab*	8.8	Cbc*	13.2	Ba	17.6	Ab	83.1	Aa*	78.9	Bb	74.5	Cc
	320 °C	7.8	Ca*	11.1	Ba	13.9	Aa	11.1	Ca*	15.3	Ba	29.9	Aa	81.1	Ab	73.6	Bc	56.1	Cd
	Reference	7.0						10.1						82.9					

Averages followed by the same capital letter between lines (time and properties) and lowercase between columns (temperature and sludge) do not differ, at 5% significance, by the Tukey test, for each type of sludge. *Do not differ from the reference, at 5% significance, by the Dunnet test, for each type of sludge.

*PS*—primary sludge, *SS*—secondary sludge, *MIX* primary and secondary sludge.

With increasing temperature and torrefaction time, an increase in ash and fixed carbon contents and a reduction in volatile matter contents of the torrefied sludge can be observed.

The contents of volatile matter of the reference PS and MIX were similar to those observed in other lignocellulosic biomass such as eucalyptus (84%) and coffee (83%)^40,41^. The fixed carbon contents were lower than those observed for eucalyptus wood (15%)^41^. The volatile matter contents of the secondary sludge were lower than those observed for the other sludges, due to its biopolymers, mainly lignin^23,39^. High contents of volatile matter result in faster burning of the material^35,42^.

The volatile matter content in the PS, SS and MIX after torrefaction was reduced by 45%, 22% and 32%, respectively, when thermal treated at 320 °C for 60 min, compared to the reference sludges. Under the same torrefaction conditions, the fixed carbon content increased by 339%, 70% and 196%. It is noteworthy that the higher the fixed carbon content in biomass, the slower will be its burning, resulting in a longer residence time of the sludge in the boiler^35,43^.

Ash content is undesirable for energy generation because it reduces the heating value, increases the frequency of ashtray cleaning and causes corrosion in biomass boiler equipment^13,42,44^. With torrefaction, the ash content increased, which was expected given the degradation of the organic fraction of the sludge. When thermal treated at a temperature of 320 °C for 60 min, the ash content in the PS, SS and MIX increased by 182%, 32% and 98%, respectively, compared to the reference sludges.

The ash content in the primary sludge possibly indicates inorganic compounds present in the plant effluent, especially the effluent from the causticization sector^16,45,46^.

The ash content observed for the secondary sludge (24.2%) was higher than the average values found in the literature (12.5%)^13^, probably due to the high cell residence time (sludge age) in the activated sludge process used in the mill^23,24^.

The average values of the elemental chemical composition (C, H, N, S and O) of the reference and torrefied sludges are presented in Table 4.

**Table 4:**
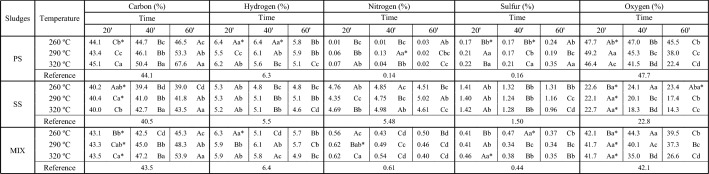
Average values of elemental chemical composition (C, H, N, S and O) of the reference and torrefied sludges.

Averages followed by the same capital letter between lines (time and properties) and lowercase between columns (temperature and sludge) do not differ, at 5% significance, by the Tukey test, for each type of sludge. *Do not differ from the reference, at 5% significance, by the Dunnet test, for each type of sludge.

*PS*—primary sludge, *SS*—secondary sludge, *MIX*—primary and secondary sludge.

The carbon content of all the sludges increased with the increase of temperature and torrefaction time, but there was a reduction in hydrogen and oxygen contents. The carbon content of the PS, SS and MIX submitted to torrefaction at 320 °C for 60 min increased about 53%, 7% and 24%, respectively, compared to the references. The oxygen contents of the sludge had reductions of 53%, 37% and 40%, respectively, compared to the reference sludges. This deoxygenation occurs mainly due to dehydration and depolymerization of the torrefied sludge^21,47^.

Evaluating the effect of the thermal treatment on the sludge, a reduction in nitrogen content and a stabilization of the sulfur content in the PS and MIX can be observed. In the secondary sludge there was no increase in these elements. It should be noted that there are already high concentrations of these elements in its composition, approximately 5% nitrogen and 1% sulfur. The presence of nitrogen contents in sludge can negatively affect its energy value, and increases emissions of harmful gases during combustion^21,36,48^.

**Figure 3:**
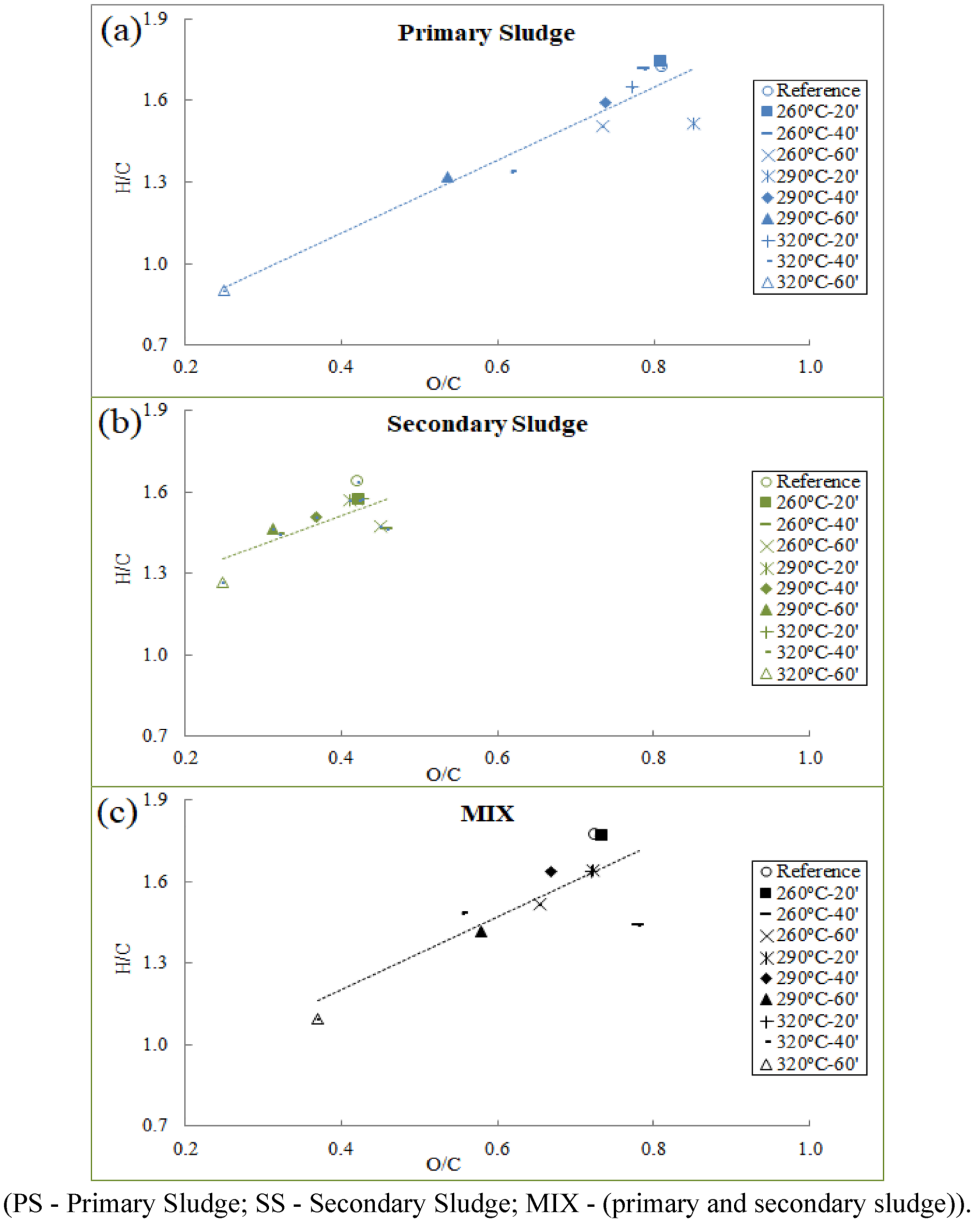
Van Krevelen diagram of the reference samples PS (**a**), SS (**b**) and MIX (**c**) and thermal treatments.

The Van Kreveken diagram is a graph of the chemical structure of the material in relation to the influence of the hydrogen to carbon (H/C) atomic ratio versus the oxygen to carbon (O/C) atomic ratio. The Van Kreveken diagram of the reference samples of PS, SS and MIX and the same 3 after thermal treatments are presented in Fig. 3.

The H/C and O/C atomic ratios of the sludges decreased with increasing torrefaction time and temperature, moving towards the lower left region. The H/C and O/C ratios of the sludge torrefied at 320 °C for 60 min were more energy efficient, showing an H/C atomic ratio closer to 1.1 and an O/C atomic ratio of 0.3, especially for the primary sludge that obtained the lowest atomic ratio, justifying its high heating value.

Usually, fuels with lower H/C and O/C ratios are classified as having a higher heating value, which helps in higher energy efficiency^21,49,50^.

The higher and lower heating values of the reference and torrefied sludges are presented in Table 5.

**Table 5:** Average values of higher and lower heating values and energy density of the reference and torrefied sludges.

Sludges	Temperature	Higher heating values (MJ kg^−1^)						Lower heating values (MJ kg^−1^)						Energy density (MJ kg^−1^)					
		Time						Time						Time					
		20′		40′		20′		40′		20′		40′		20′		40′		20′	
PS	260 °C	17.5	Bc	17.6	Bc	17.9	Ac	15.3	Cc	15.8	Bc	16.4	Ac	2.53	Aa*	2.37	Bb	2.24	Cc
	290 °C	17.7	Cb	18.1	Bb	19.7	Ab	16.0	Cb	16.4	Bb	18.1	Ab	2.55	Aa*	2.41	Bb	2.55	Aa*
	320 °C	18.1	Ca	19.5	Ba	25.5	Aa	16.4	Ca	17.8	Ba	24.0	Aa	2.44	Bb	2.58	Aa*	2.48	Bb
	Reference	17.2						13.7						2.55					
SS	260 °C	17.6	Ba	17.5	Bb	17.7	Ac	15.8	Cab	16.0	Bb	16.5	Ac	9.52	Ba	9.51	Bb	9.84	Ac
	290 °C	17.4	Cb	17.7	Ba	18.0	Ab	15.7	Cb	16.4	Ba	16.8	Ab	9.34	Ca	9.62	Ba	9.95	Ab
	320 °C	17.4	Cab	17.8	Ba	18.6	Aa	15.8	Ca	16.4	Ba	17.5	Aa	9.32	Ca	9.61	Ba	10.27	Aa
	Reference	17.2						13.8						8.15					
MIX	260 °C	17.1	Cb	17.3	Bc	17.6	Ac	15.3	Cc	16.0	Bc	16.2	Ac	2.96	Bb*	3.09	Ac	2.95	Bc*
	290 °C	17.1	Cbc*	17.7	Bb	18.2	Ab	15.5	Cb	16.1	Bb	16.8	Ab	2.97	Cb	3.16	Bb	3.30	Aa
	320 °C	17.4	Ca	18.2	Ba	20.1	Aa	15.8	Ca	16.6	Ba	18.8	Aa	3.06	Ba	3.29	Aa	3.22	Ab
	Reference	17.0						13.4						2.93					

Averages followed by the same capital letter between lines (time and properties) and lowercase between columns (temperature and sludge) do not differ, at 5% significance, by the Tukey test, for each type of sludge. *Do not differ from the reference, at 5% significance, by the Dunnet test, for each type of sludge.

*PS*—primary sludge, *SS*—secondary sludge, *MIX*—primary and secondary sludge.

The higher heating values of the reference sludges were similar to that of urban ETP sludge (17.5 MJ kg^−1^)^51^, and also to that of other lignocellulosic biomasses that are used for energy, such as coffee stem waste (17.7 MJ kg^−1^)^52^, sugarcane bagasse (17.4 MJ kg^−1^)^53^ and soybean wastes (bark, stalk and defective grains) (16.7 MJ kg^−1^)^54^. The data obtained in other studies encourages the incineration of sludge in boilers that have high potential for energy production in mills^55^.

The heating value of the sludges increased with the increase of temperature and torrefaction time. The treatment with torrefaction at 320 °C for 60 min had a significant increase of 48%, 8% and 18% compared to the reference, respectively, for PS, SS and MIX.

As presented in Tables 3 and 5, the loss of volatile matter and the degradation of less thermally stable lower heating value constituents may be the main reason for the energy gains with increasing torrefaction temperature and time. Consequently, there is an increase in fixed carbon as a result of the concentration of lignin in the sludge^13,18,19,37^.

In general, it is noted that the secondary sludge had lower increases in heating value with torrefaction. This may have occurred due to the higher ash content and lower carbon and hydrogen content in its composition, which negatively influence the energy generation of any biomass^53,56^.

The increase in LHV from sludge torrefaction is desirable because the process reduces the moisture content and atomic O/C ratio^21,50,52^.

Following the same trend as HHV, the LHV of the sludge increased with the increase of temperature and time of torrefaction. The sludge torrefied at a temperature of 320 °C for 60 min obtained increases of 76%, 27% and 41% over the reference, for PS, SS and MIX, respectively.

The torrefied SS had lower increases in LHV when compared to the PS and MIX. The secondary sludge presented high H/C and O/C atomic ratios and high nitrogen contents that can contribute to the formation of undesirable nitrogen compounds, such as NO_x_, N_2_O and HCN, reducing the energy efficiency of the process^21,40,57^.

Sludge with low moisture content can be considered a potential fuel source, but as mentioned earlier, the high moisture content can leave the energy gain nil or even negative^13,37,55,58^.

The energy density indicates the energy potential of the sludge, in units of energy per volume. There was an effect of the thermal treatments on the energy density of the sludges evaluated. An increase in energy density occurred from 8.2 and 2.9 GJ m^−3^ to 10.3 and 3.2 GJ m^−3^ of the reference for the SS and MIX, respectively, submitted to torrefaction at 320 °C for 60 min. The sludge torrefied at 320 °C for 60 min had significant increments of 26 and 10% over the reference, for the SS and MIX, respectively.

The primary sludge torrefied at a temperature of 320 °C for 60 min, on the contrary, obtained a decrease of 3% in energy density compared to the reference. This fact was due to its physicochemical characteristic of a higher concentration of sugars, such as hemicelluloses, which is the less thermally stable fraction and consequently more degraded during torrefaction^9,21^.

Torrefaction significantly increases the energy density of the sludge, and thus is a technically feasible method for eliminating some of the disadvantages of raw sludge for energy purposes, because it reduces transportation and storage costs, since it contains a greater amount of energy per unit volume^49,59^.

The energy yield of the sludges decreased with increasing torrefaction temperature and time, showing the lowest performance when torrefied at 320 °C for 60 min. The energy yield of the sludge ranged from 99 to 72% for the temperature range of 260–320 °C.

The energy yield of sludges decreases during torrefaction, mainly due to the higher degradation of hemicelluloses, a less thermally stable fraction, while cellulose and lignin degrade only partially, being thermally more stable at the temperatures used in this experiment^13,21,50^.

This is one of the great advantages of the sludge torrefaction process, eliminating low-energy materials and enhancing its energetic characteristics for combustion in biomass boilers in the kraft pulp mills themselves.

## Conclusions

The present study presented the technical feasibility of transforming primary, secondary and mixed (primary and secondary) sludges generated in the effluent treatment plant of kraft pulp mills into torrefied sludge for energy generation. This research plays a key role in promoting the circular economy, Sustainable Development Goals (SDGs) and Environmental, Social and Governance (ESG) criteria.

Increasing the torrefaction temperature and residence time of the primary, secondary and mixed sludges, several improvements in their physical and chemical properties were observed. These include an increase in heating value by eliminating less energetic compounds and concentrating the fixed carbon content, a reduction in moisture content leading to a higher lower heating value, and high energy yield and increased energy density, which are crucial factors for sludges intended for energy utilization.

The treatment using 320 °C for 60 min showed the best energy efficiency for the sludges. The primary sludge, in relation to the other sludges, was the one that stood out the most after the thermal treatments for energy generation.

This research not only supports the principles of the circular economy but also aligns with Sustainable Development Goals (SDGs) and Environmental, Social, and Governance (ESG) criteria. It contributes to SDG 7 (Clean and Affordable Energy) and SDG 12 (Responsible Consumption and Production) by offering cleaner energy sources and promoting sustainable practices.

From an ESG perspective, the conversion of sludge into torrefied biomass reduces greenhouse gas emissions, enhances waste management, and utilizes renewable energy. Process optimization ensures operational efficiency, safety, and compliance with environmental regulations.

The transformation of sludge into torrefied biomass for energy generation presents an innovative and sustainable solution with potential benefits for the Brazilian bleached kraft pulp industry, including environmental preservation, greenhouse gas emission reduction, and socioeconomic development. However, addressing challenges related to commercial-scale implementation remains essential, requiring further research to improve technical and economic feasibility. The findings of this research can help overcome this industry bottleneck.

## Data Availability

The datasets generated during and/or analysed during the current study are available from the corresponding author on reasonable request.
